# Is Dandruff a Disease?

**DOI:** 10.4103/0974-7753.66922

**Published:** 2010

**Authors:** Frederick Manuel

**Affiliations:** Adjunct Professor of Dermatology, Department of Dermatology and STD, Sri Ramachandra Medical College and Hospital, Porur, Chennai – 600 116, India

Sir,

Dandruff (*Pityriasis capitis*) is a non-inflammatory form of seborrheic dermatitis, with increased scalp scaling, which represents the more active end of the spectrum of physiological desquamation, while seborrheic dermatitis (Pityrosporal dermatitis, dermatitis of the seborrheic areas) is an inflammatory, erythematous, scaly eruption that occurs primarily in ‘seborrheic’ areas, that is, mainly in areas with a high number and activity of sebaceous glands, such as, scalp, central areas of the face, and upper trunk. This ‘disease’ (Seborrheic dermatitis) is one of the accelerated epidermal growths resulting in retention of nuclei that have not had sufficient time to completely mature, in the cells of the stratum corneum.[1] As seborrheic dermatitis is considered a disease, then dandruff too should be considered a disease as it is only a non-inflammatory form of seborrheic dermatitis.

A disease is defined as an abnormal condition of an organism that impairs bodily functions associated with specific signs and symptoms (Dorland’s Medical Dictionary). In human beings, ‘disease’ is often used more broadly to refer to any condition that causes discomfort, dysfunction, distress, social problems, and death to the person afflicted or similar problems for those in contact with the person. By this definition, dandruff is also a disease.

Malassez, (1874) proposed that yeast was the etiological agent in seborrheic dermatitis. Malassezia was increased 1.5 to 2 times (75% of flora) in dandruff.

The critical level of colonization with the yeast leads to the release of pro-inflammatory mediators. This further leads to subclinical microinflammation, which finally results in dandruff. Dandruff is dander and dander represents nothing more than a physiological scale. Therefore, dandruff is considered a physiological process, calling for cosmetic management.[2]

Dandruff has recently been considered as the most commercially exploited skin and scalp disorder / disease by the personal care industry[3]

Therefore, finally, is dandruff a disease? It most probably is. Consider the following.

First, one school of thought seems to think that it is only a physiological scale. However, in reality, scalps of patients with dandruff liberate up to about 800,000 cells/sq cm compared to only about 500,000 cells/sq cm for the normal scalp. Furthermore, in dandruff there are about 25,000 nucleated cells/sq. cm compared to about 3,700 nucleated cells/sq cm on the normal scalp.

Second, as seborrheic dermatitis is indisputably a disease, then dandruff, which is considered a non-inflammatory form of seborrheic dermatitis, should also be considered a disease. Also, although there is no inflammation clinically, there is a pathological, subclinical, microinflammation taking place.

Third, Malassezia furfur, the etiologic agent agent implicated in dandruff and seborrheic seems to fulfill all Koch’s postulates regarding dandruff and seborrheic dermatitis.

Finally, dandruff qualifies well as a disease according to its definition.

## References

[1] Wattanakrai P, Arndt KA, Hsu JT (2007). Seborrheic dermatitis and dandruff. Manual of dermatologic therapeutics.

[2] Piérard-Franchimont C, Xhauflaire-Uhoda E, Piérard GE (2006). Revisiting dandruff. Int J Cosmet Sci.

[3] Ranganathan S, Mukhopadhyay T (2010). Dandruff: The most commercially exploited skin disease. Indian J Dermatol.

